# Supplementary material for: Positive Association between Preserved C-Peptide and Cognitive Function in Pregnant Women with Type-1 Diabetes
Source: Biomedicines. 2022 Nov 2;10(11):2785. doi: 10.3390/biomedicines10112785 (PMC9687841; doi:10.3390/biomedicines10112785)
Supplement: Supplementary file 1 [file biomedicines-10-02785-s001.zip › biomedicines-1943053-supplementary.pdf]

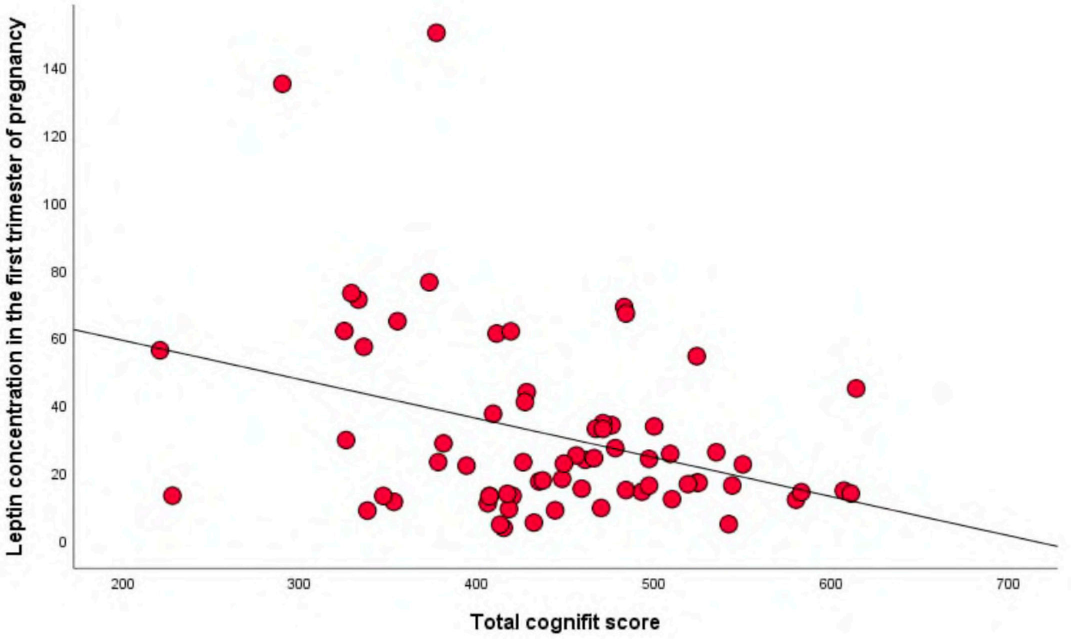

Figure S1. Linear correlation between total cognitive function (score) and leptin concentration in the first trimester of pregnancy (*r* = - 0.349, *p* = 0.001).

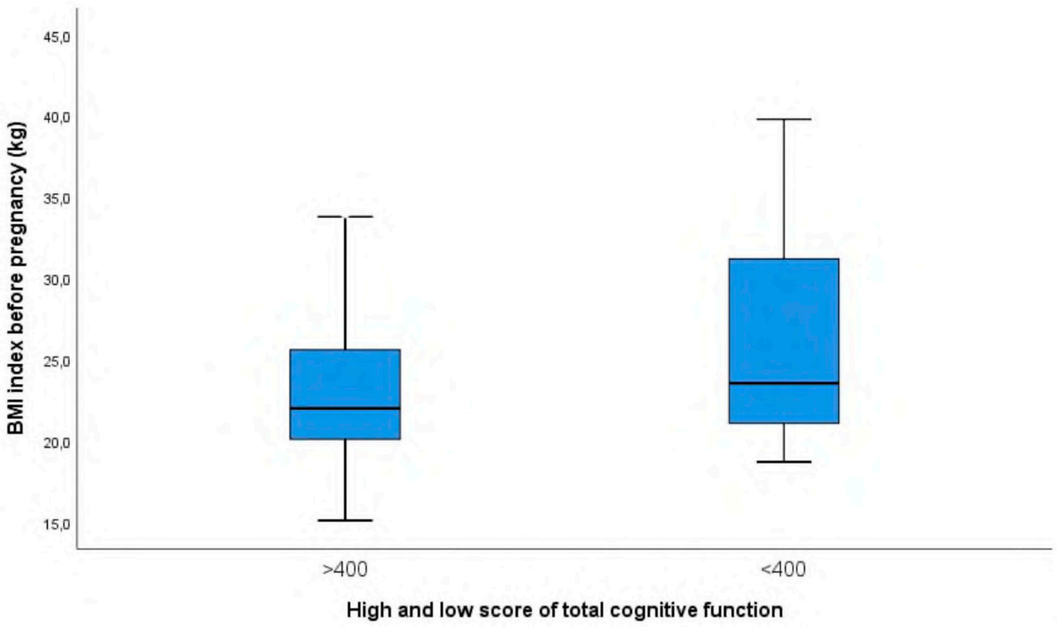

Figure S2. Presentation of mean values with SD of BMI in groups of pregnant women with a higher score (23.4 ± 4.7 kg/m^2) and with a low score (26.2 ± 5.7 kg/m^2) for total cognitive function.

Table S1. Characteristics of studies used in the meta-analysis.

		Cognitive score >400	Cognitive score <400
Age (years)	<30	23	7
	>30	35	13
Age of onset of T1DM (years)	Before 10	21	7
	After 10	37	13
Duration T1DM (years)	<8	18	5
	>8	40	15
BMI (kg/m^2)	<25	42	11
	>25	13	12
HbA1c in 1st trimester (%)	<6.5	25	11
	>6.5	28	9
Hypothyroidism	NO	32	4
	YES	22	16
C-peptide 1st trimester (pmol/L)	YES	35	7
	NO	2	11
CAN	NO	35	7
	YES	20	12
Education	High and University. degree	26	10
	High school	28	14
CGM > 7.8 mmol/L	<7.8	36	10
	>7.8	12	7
CGM < 3.9 mmol/L	<3.9	12	7
	>3.9	36	10
